# Coagulation is associated with renal function decline and chronic kidney disease in people living with HIV in South Africa

**DOI:** 10.3389/fneph.2026.1800778

**Published:** 2026-05-22

**Authors:** Joel Choshi, Haskly Mokoena, Sihle E. Mabhida, Brian T. Flepisi, Helen C. Steel, Kabelo Mokgalaboni, Wendy N. Phoswa, Gerald Maarman, Bongani B. Nkambule, Phiwayinkosi V. Dludla, Sidney Hanser

**Affiliations:** 1 Department of Physiology and Environmental Health, University of Limpopo, Sovenga, South Africa; 2 Shanghai Key Laboratory of Orbital Diseases and Ocular Oncology, Health Systems Research Unit, South African Medical Research Council, Tygerberg, South Africa; 3 Department of Pharmacy and Pharmacology, University of the Witwatersrand, Braamfontein, Johannesburg, South Africa; 4 Department of Immunology, University of Pretoria, Pretoria, South Africa; 5 Department of Life and Consumer Sciences, College of Agriculture and Environmental Sciences, University of South Africa, Roodepoort, South Africa; 6 Centre for Cardio-Metabolic Research in Africa (CARMA), Division of Medical Physiology, Department of Biomedical Sciences, Faculty of Medicine & Health Sciences, Stellenbosch University, Cape Town, South Africa; 7 School of Laboratory Medicine and Medical Sciences, University of KwaZulu-Natal, Durban, South Africa; 8 Department of Biochemistry and Microbiology, University of Zululand, KwaDlangezwa, South Africa; 9 Department of Physiology, School of Medicine, Sefako Makgatho Health Sciences University, Tshwane, South Africa

**Keywords:** antiretroviral therapy, chronic kidney disease, coagulation, C-reactive protein, D-dimer, eGFR_cystC_, fibrinogen, inflammation

## Abstract

**Background:**

Chronic inflammation is a well-documented feature of human immunodeficiency virus (HIV) infection and is implicated in the progression of several comorbidities, including chronic kidney disease (CKD). Notably, chronic coagulation activation is another common phenomenon mediated by chronic inflammation among people living with HIV (PLWH) and has also been linked to CKD progression. However, the relationship between inflammation, coagulation and renal dysfunction remains underexplored in African populations with high HIV prevalence. This study investigated the association between systemic inflammation, coagulation activation and renal function among PLWH, particularly those receiving antiretroviral therapy (ART).

**Methods:**

In this cross-sectional study, 151 adults were enrolled, comprising 80 PLWH on ART, 27 PLWH not on ART, and 44 people not living with HIV (PNLWH). Inflammatory marker, C-reactive protein (CRP) and coagulation markers including fibrinogen and D-dimer, were measured and CKD stages were determined using estimated glomerular filtration rate (eGFR), which was calculated based on cystatin C (cystC) levels (eGFR_cystC_).

**Results:**

In the overall PLWH cohort, D-dimer was significantly associated with reduced eGFR_cystC_ in both crude (*p* = 0.004) and adjusted analyses (*p* = 0.014), indicating poorer kidney function. Fibrinogen showed a significant association with reduced eGFR_cystC_ only after adjusting for confounders (*p* = 0.048). Among PLWH on ART, D-dimer remained independently associated with reduced eGFR_cystC_, while fibrinogen showed an independent association after adjustment (β = -1.54, *p* = 0.031). Elevated fibrinogen levels were associated with an increased odds of CKD in the overall PLWH group (AOR = 1.12, 95% CI 1.00–1.23, *p* = 0.0 41), and elevated D-dimer levels were associated with increased odds of CKD among those on ART (AOR = 1.01, 95% CI 1.00–1.03, *p* = 0.045).

**Conclusion:**

The study reveals that both D-dimer and fibrinogen, key markers of coagulation are independently associated with renal function decline and risk of CKD in PLWH, particularly those on ART. These findings suggest that coagulation activation may be an early indicator of declining kidney function and increased risk of CKD in PLWH, potentially preceding overt systemic inflammation.

## 1 Introduction

Renal dysfunction is now considered a major complication and fourth leading cause of death globally in people living with HIV (PLWH) (1–3). The burden of renal dysfunction remains markedly higher in low-middle income countries than in high-income countries (4). This condition is classified into two categories: acute kidney injury (AKI) and chronic kidney disease (CKD). Chronic kidney disease is the most common type and disproportionately affects Africans, with an estimated threefold higher chance of developing in PLWH compared to the general population (3, 4). The overall CKD prevalence among PLWH in Africa was estimated to be 7.9%. Moreover, the lowest prevalence was reported in South Africa at 1.9% and highest prevalence in Nigeria at 19.3% (4). The high prevalence of CKD in PLWH is attributed to a combination of factors, including antiretroviral therapy (ART)-related nephrotoxicity, HIV-specific mechanisms (HIV-associated nephropathy, immune activation or inflammation) and co-morbidities like diabetes or hypertension, as well as co-infections such as hepatitis B or C (3, 5).

An abnormal immune response, typically characterized by elevated pro-inflammatory markers, is already recognized as a prominent feature contributing to renal damage in PLWH (6, 7). Indeed, chronic immune activation and inflammation are widely recognized pathological features in PLWH, even in those who are successfully maintained on ART, and leads to coagulation pathway activation, creating a prothrombotic environment that further promotes or enhances inflammation (8–11). C-reactive protein (CRP), fibrinogen and D-dimer are some of the well-recognized inflammatory and coagulation molecules associated with renal function decline in PLWH (12–18). Emerging evidence suggest that coagulation activation may drive endothelial dysfunction and microvascular injury, contributing to impaired renal perfusion and glomerular damage (11, 19, 20). Markers such as D-dimer and fibrinogen reflect thrombin activation and fibrinolysis, processes increasingly recognized in HIV-associated renal pathology (10, 19, 21).

Literature continues to indicate that CRP and fibrinogen are well-recognized inflammatory and coagulation biomarkers that can be estimated in blood samples, with CRP reflecting acute-phase inflammation and fibrinogen playing a key role in detecting a state of coagulation that is linked to renal function decline in PLWH (4, 22, 23). Although available data highlights the importance of CRP, fibrinogen and D-dimer as potential biomarkers in PLWH (16, 24, 25), such data remains scant within populations in South Africa, where the prevalence of renal dysfunction is a health concern (26). The study reported a CKD prevalence of 7% in PLWH, which was associated with age and diabetes (26). Thus, it is important to establish whether raised inflammatory response and coagulation is consistent with renal dysfunction in PLWH, especially in the South African context, where the HIV disease burden is high (27–30) and data is generally lacking. The HIV prevalence in South Africa increased from 13–17.8% between 2000 and 2022 in those aged 15–49 years, with 8.5 million people currently living with HIV in the country (31).

The current hypothesis suggests that persistent immune activation may contribute to cellular damage through the persistent elevation of inflammatory and coagulation markers beyond other pathological mechanisms in PLWH (32, 33). Moreover, ART itself can modulate inflammatory responses that may subsequently lead to kidney injury due to drug-specific toxicities (34). Coagulation activation contributes to kidney injury and renal disease progression due to the resulting blood clots restricting blood flow to the kidneys, leading to tissue damage and organ dysfunction (35–37). While established renal biomarkers such as creatinine are essential for monitoring kidney health (38), growing evidence suggests they may lack the sensitivity to detect the subtle, ongoing renal function decline often observed in PLWH (39, 40). Creatinine is often affected by several factors in PLWH, including age, dietary intake, muscle mass, ART effects and HIV-associated muscle wasting. Substantial evidence highlights the use of cystatin C as an alternative marker to creatinine as it is less affected these factors, ultimately offering a more accurate estimation of glomerular filtration rate in PLWH compared to creatinine (41). This study investigated the association between systemic inflammation, coagulation activation and renal function among PLWH, particularly those on ART. We hypothesized that coagulation activation, more than systemic inflammation, would be independently associated with renal function decline and increased risk of CKD among PLWH.

## 2 Study methods

### 2.1 Study design and population

This is a cross-sectional study of 151 adult participants comprising PLWH on antiretroviral therapy (ART), PLWH not on ART, and people not living with HIV (PNLWH) visiting the Mankweng Hospital located in Limpopo Province, South Africa. A cross-sectional design was employed to evaluate associations rather than causality, given logistical constraints and the lack of longitudinal follow-up. The study was conducted from April 2023 to December 2024 (~ 20 months). The study model is validated and based on published literature (26, 42). The sample size was predetermined using the Cochran formula (43) to obtain the ideal sample size, with a 5% margin of error, 95% confidence level and HIV prevalence of 11.8% for Limpopo Province (30). The calculation is described below. Adults aged ≥18 years were eligible for participation. Participants were required to be either people living with HIV (PLWH), with or without antiretroviral therapy (ART), or HIV-negative controls. The study was restricted to adults (≥18 years) to minimize heterogeneity related to developmental, physiological, and immunological differences observed in pediatric populations, which may influence inflammatory, coagulation, and renal markers. Patients previously diagnosed with renal disease and having co-infections such as *Mycobacterium tuberculosis* or hepatitis virus B and C as well as pregnant or breastfeeding women, were excluded at the time of enrolment based on their medical history. Exclusion also extended to those with co-morbidities such as diabetes and hypertension, and those taking other medications such as cimetidine or nonsteroidal anti-inflammatory drugs that can potentially affect the study outcomes.


$$n=\frac{{\left(t\right)}^{2}\;X\;p(1-p)}{{\left(m\right)}^{2}}$$

n is the calculated sample size.
t is a confidence level at 95% (standard value of 1.96).
p is the estimated prevalence of HIV in Limpopo (0.118).
m is the margin of error at 5% (standard value of 0.05).

$$\begin{array}{c} n=\frac{{\left(1.96\right)}^{2}\;X\;0.118(1-0.118)}{{\left(0.05\right)}^{2}}\\ =152.59 \end{array}$$

### 2.2 Data collection

This study was approved by the University of Limpopo Ethics Committee (project number TREC/105/2023: PG) and was conducted following the principles of the Declaration of Helsinki (44). Participants’ demographics (including age and sex), lifestyle factors (such as smoking status), blood pressure, anthropometry data (including body mass index and waist-to-hip ratio), and clinical data such as ART or medication use, type of ART regimen and specific duration were collected at the time of enrolment. The medical records were checked to ensure that all the data given is accurate. Participants’ blood pressure was measured using an Omron M2 monitor (OMRON Healthcare, Kyoto, Japan) following the manufacturer’s instructions. Blood pressure was measured in the morning in a quiet room when the participant was relaxed and in minimal clothing. Measurements were taken on the right arm, ensuring the arm cuff was applied correctly and that the air tube was positioned on the side of the elbow, connected to the main unit. Two measurements were taken at five-minute intervals, and the average was calculated.

Blood samples were collected from all participants by a qualified health professional. The procedure entailed withdrawing 5 milliliters of venous blood from the median cubital vein into serum tubes and ethylenediaminetetraacetic acid-containing vacutainer tubes (Beckton Dickinson, Franklin Lakes, NJ, USA). Standard operating procedures for specimen collection and handling were adhered to. Blood samples were processed to obtain serum and plasma fractions through centrifugation using an Allegra X-30 Series benchtop centrifuge, which was calibrated prior to processing (Beckman Coulter, Brea, CA, USA). To verify the disease status of the participants, all blood samples were tested for HIV by researchers using the Alere Determine HIV-1/2 kit (Alere to Abbott Medical Co Ltd., Tokyo, Japan), strictly adhering to manual protocol.

The CD4+ T-cell count was determined for all PLWH using the Alere PIMA analyzer (Alere Technologies GmbH, Jena, Germany), guided by the manufacturer’s instructions. The Alere PIMA analyzer was calibrated, and quality controls were done to ensure proper functionality and accurate results. The procedure was performed under expert supervision. Anthropometric parameters included weight, height, body mass index (BMI), waist circumference, and hip circumference. Weight was measured using an accurate reading digital body weight scale (Pee Pee Electricals, Delhi, India) while height was measured using a portable Seca 213 stadiometer (Seca, Hamburg, Germany) as per the manufacturer’s instructions, following standard measuring procedures. Anthropometry and blood pressure measurements were performed by well-trained researchers following established standard protocols. Body mass index (BMI) was calculated as weight in kilograms (kg) divided by the square of the height in meters (m^2^), while waist-to-hip ratio (WHR) was calculated as waist circumference divided by hip circumference.

### 2.3 Biochemical analysis, including renal markers, glucose levels, and lipid profile

Markers of inflammation (serum CRP), coagulation (serum fibrinogen and D-dimer) and renal function (plasma cystatin C) were all measured by bead-based multiplex immunoassay using Luminex^®^ xMAP^®^ technology (Millipore Sigma, Darmstadt, Germany) guided by the manufacturer’s instructions. Three types of kits were employed, the human Cardiovascular Disease (CVD) (acute phase) magnetic bead panel 3 for serum CRP and fibrinogen (catalogue number: HCVD3MAG-67K, Millipore Sigma, Darmstadt, Germany), human CVD magnetic bead panel 2 - cardiovascular disease multiplex assay for D-dimer (catalogue number: HCVD2MAG-67K, Millipore Sigma, Darmstadt, Germany) and the human kidney injury magnetic bead panel 6 for plasma cystatin C (catalogue number: HKI6MAG-99K, Millipore Sigma, Darmstadt, Germany).

This was followed by determining the estimated glomerular filtration rate (eGFR) employing the chronic kidney disease-epidemiology collaboration (CKD-EPI) formula (45). Cystatin C-based estimated glomerular filtration rate (eGFR_cystC_) was categorized into stages including normal renal function (eGFR_cystC_ of ≥ 90 mL/min/1.73m^2^), mild renal dysfunction (eGFR_cystC_ of 60–89 mL/min/1.73m^2^) and moderate-to-severe renal dysfunction and renal failure, also known as chronic kidney disease (CKD) (eGFR_cystC_ of < 60 mL/min/1.73m^2^). The National Kidney Foundation Kidney Disease Outcomes Quality Initiative (NKF KDOQI) guidelines were used (46).

Serum glucose and lipid levels, including high-density lipoprotein cholesterol (HDL-c), total cholesterol, and triglycerides, were analyzed using the Cobas Integra^®^ 400 Plus autoanalyzer (Roche Holding AG, Indianapolis, IN, USA) as per the manufacturer’s instructions. Low-density lipoprotein cholesterol (LDL-c) results were obtained from the Laboratory information system. Diabetes and dyslipidaemia have a profound impact on renal function (47), hence, the importance of including glucose and lipid profiles in the current study to adjust for these confounders.

All methods and instruments were validated prior to and during data collection to ensure the accuracy and reliability of the data. Procedures including ELISA and multiplex immunoassays were performed under the supervision of an expert. The equipment or instruments used, including Luminex analyzer and chemistry autoanalyzer, were all calibrated and internal quality controls were done to ensure proper functionality and accurate results. Luminex technology is a highly efficient and accurate platform, equipped with quality control programs that monitor precision and accuracy. The standard curves of each analyzed across kit lots was checked and validated, and there was a rigorous control of sample preparation. The blood samples were run in duplicates to ensure quality control, assess variance, and enhance accuracy. Similarly, the Cobas Integra 400 Plus is designed for precision and accuracy due to its quality control programs. Verified estimating formulas and standard classification criteria were used. See **Supplementary file S2** (Quality Assurance Procedures for Biochemical Analysis).

### 2.4 Statistical analysis

Data was captured and analyzed on IBM Statistical Package for Social Sciences (SPSS) statistics software version 30 (International Business Machines Corporation, Armonk, New York, USA). A Kolmogorov test of normality was performed to determine whether the data follows a normal or non-normal distribution. A one-way analysis of variance (ANOVA) was employed for variables with normal distribution while a Kruskal-Wallis test was performed for variables with non-normal distribution. *Post hoc* analyses were performed using Bonferroni correction following ANOVA and pairwise comparisons following the Kruskal–Wallis test to identify group-specific differences. Categorical variables were summarized as frequencies and percentages, and differences between groups were assessed using the chi-square test. The associations between inflammation, coagulation and renal function were assessed using the Spearman correlation analysis, linear regression, and binary logistic regression. Linear regression was not performed for PNLWH due to the limitation of the small sample size for this cohort. A *p*-value of < 0.05 was considered as statistically significant for differences and associations.

## 3 Results

A total of 151 participants were included in the study. Overall, coagulation markers showed stronger associations with renal function than inflammatory markers. D-dimer levels were significantly elevated in PLWH, particularly those on ART, and increased progressively with worsening renal function. In contrast, CRP showed no significant association with renal function across study groups. Fibrinogen and D-dimer were independently associated with reduced eGFR_cystC_ in PLWH, with D-dimer demonstrating consistent associations in both crude and adjusted analyses, especially among those on ART. Furthermore, elevated coagulation markers were associated with increased odds of chronic kidney disease (CKD), whereas CRP was not a significant predictor.

### 3.1 Demographic and clinical characteristics of the study population

A total of 151 participants (≥ 18 years), including 80 PLWH on ART, 27 PLWH without ART, and 44 PNLWH, were enrolled in the study. The demographics and clinical characteristics, including age, sex, smoking status, ART use, CD4+ T-cell count, BMI, blood pressure, glucose and lipid profile among the groups are presented in **Table 1**. The results show that age was significantly higher in PLWH on ART [42.50 years (IQR 36.25 – 49.75)] compared to PNLWH [32 years (IQR 25.00 – 45.50)] (*p* = 0.001) (**Table 1**). Females comprised 56%, 59.30% and 65.90% of PLWH on ART, PLWH without ART, and PNLWH, respectively (**Table 1**). Sex and cigarette smoking showed no significant difference among the three study groups (**Table 1**). The majority of PLWH on ART were on treatment duration of ≥ 3 years (62.70%), and five participants had missing ART duration information (**Table 1**).

**Table 1:** Demographic and clinical characteristics of the study population.

			PLWH on ART *N* = 80	PLWH without ART *N* = 27	PNLWH *N* = 44	*p*-value
Age (years)			42.50 (36.25–49.75)	38.00 (28.00–45.00)	32 (25.00–45.50)	< 0.001
Sex, *n* (%)	Male		24.00 (30.00)	11 (40.70)	15 (34.10)	0.583
	Female		56.00 (70.00)	16 (59.30)	29 (65.90)	
Cigarette smoking, *n* (%)	No		60.00 (75.00)	21.00 (77.80)	37.00 (84.10)	0.503
	Yes		20.00 (25.00)	6.00 (22.20)	7.00 (15.90)
ART use, *n* (%)			80.00 (100)	–	–	–
Duration on ART, months			53.00 (15.00–93.00)	–	–	–
Duration on ART		< 3 years	28.00 (37.30)	–	–	–
		≥ 3 years	47.00 (62.70)	–	–
CD4+ T-cell count (cells/µL)			442.60 ± 216.74	319.22 ± 284.72	–	0.030
BMI (kg/m^2^)			26.12 (22.03–30.87)	23.27 (20.49–26.67)	27.00 (22.28–32.81)	0.043
Waist-to-hip ratio			0.85 (0.80–0.90)	0.85 (0.79–0.91)	0.85 (0.79–0.90)	0.948
Systolic blood pressure (mmHg)			121.43 ± 20.27	118.07 ± 14.25	117.95 ± 14.13	0.496
Diastolic blood pressure (mmHg)			75.85 ± 10.02	73.19 ± 9.63	74.18 ± 8.75	0.391
Glucose (mmol/L)			5.20 (4.60–5.80)	4.70 (4.40–5.20)	4.80 (4.40–5.58)	0.069
Total cholesterol (mmol/L)			4.35 (3.73–5.18)	3.40 (2.90–4.10)	4.15 (3.60–4.88)	< 0.001
Triglycerides (mmol/L)			1.16 (0.88–1.46)	1.20 (0.90–1.50)	1.07 (0.80–1.35)	0.489
CRP (ng/mL)			0.88 (0.24–3.42)	0.90 (0.28–2.67)	0.685 (0.14–1.23)	0.180
Fibrinogen (ng/mL)			0.17 (0.07–0.40)	0.41 (0.17–1.00)	0.14 (0.05–0.40)	0.017
D-dimer (ng/mL)			41.54 (17.10–176.92)	17.50 (2.19–57.80)	15.18 (3.69–96.32)	0.007

Age is significant for PLWH on ART versus PNLWH (adjusted *p* = 0.001). BMI is significant for PLWH without ART versus PNLWH (adjusted *p* = 0.039). Total cholesterol is significant for PLWH on ART versus PLWH without ART (adjusted *p* < 0.000) and for PLWH without ART versus PNLWH (adjusted *p* = 0.006). Fibrinogen is significant for PLWH on ART versus PLWH without ART (adjusted *p* = 0.025) and for PLWH without ART versus PNLWH (adjusted *p* = 0.031). D-dimer is significant for PLWH on ART versus PLWH without ART (adjusted *p* = 0.037) and for PLWH on ART versus PNLWH (adjusted *p* = 0.027). ART, antiretroviral therapy; BMI, body mass index; CD4+, cluster of differentiation 4; CRP, C-reactive protein; HIV, human immunodeficiency virus. PLWH, people living with HIV; PNLWH: people not living with HIV. results expressed as median (interquartile range) for non-Gaussian variables and as mean ± standard deviation for Gaussian variables. *p*-values in bold indicate significance.

**Figure 1:**
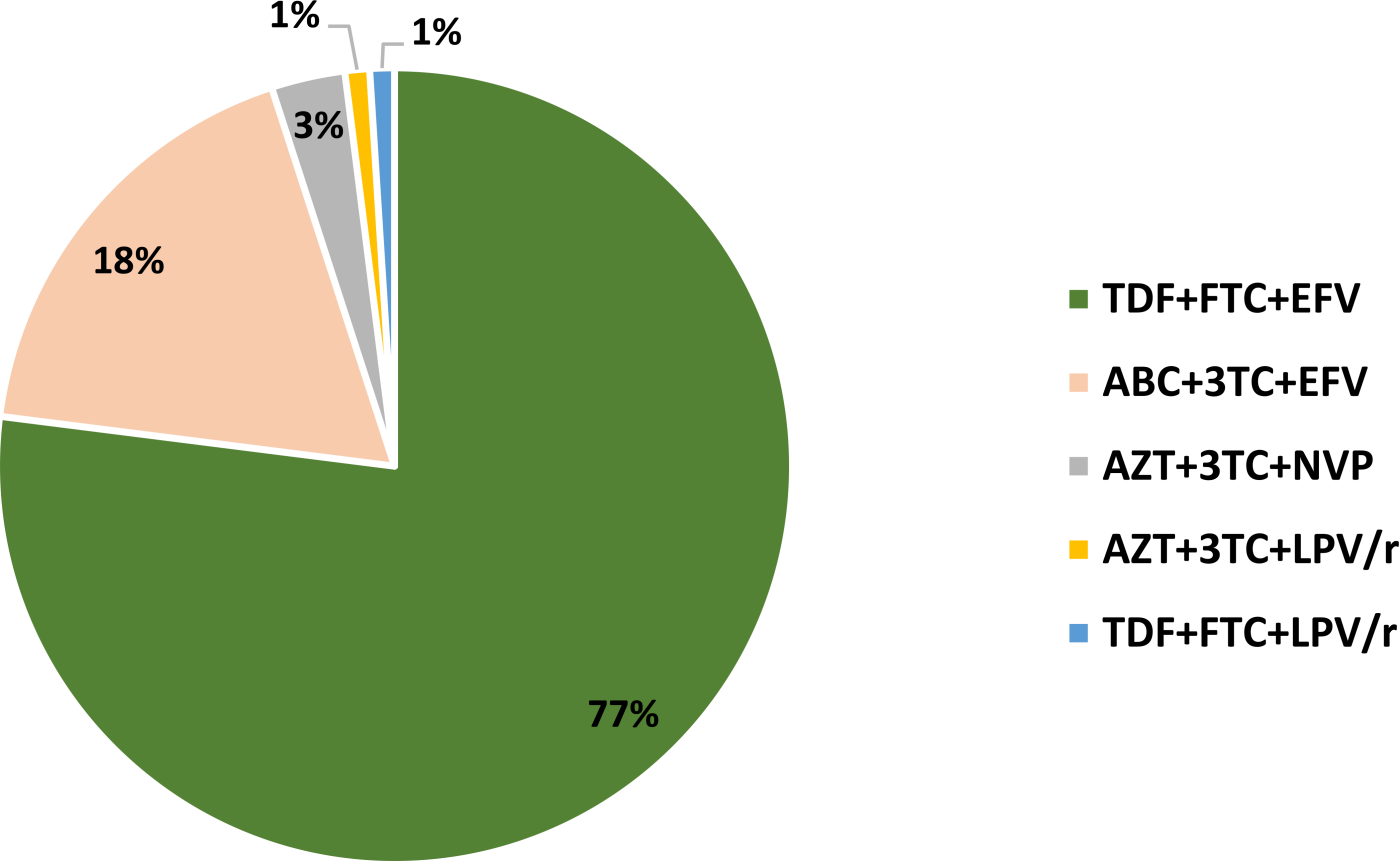
Distribution of antiretroviral therapy combinations. 3TC, lamivudine, ABC, abacavir; AZT, zidovudine; EFV, efavirenz; FTC, emtricitabine; LPV/r, ritonavir-boosted lopinavir; NVP, nevirapine; TDF, tenofovir disoproxil fumarate.

Moreover, most PLWH on ART were receiving a combination of tenofovir disoproxil fumarate (TDF) + emtricitabine (FTC) + efavirenz (EFV) (77%), followed by zidovudine (AZT) + lamivudine (3TC) + ritonavir-boosted lopinavir (LPV/r) (18%), AZT + 3TC+ nevirapine (NVP) (3%), abacavir (ABC) +3TC+EFV (1%) and TDF+FTC+LPV/r (1%) (**Figure 1**). There was a significant difference in the CD4+ T-cell count between PLWH on ART and those without ART (*t* (94) = 2.20, *p* = 0.030), with it being significantly higher in those on ART (442.60 ± 216.74 cells/µL) compared to those without ART (319.22 ± 284.72 cells/µL) (**Table 1**). The BMI was significantly lower in PLWH without ART [23.27 kg/m^2^ (IQR 20.49 – 26.67)] compared to PNLWH [27.00 kg/m^2^ (IQR 22.28 – 32.81)] at adjusted *p* = 0.039 (**Table 1**). The total cholesterol was significantly higher in PLWH on ART [4.35 mmol/L (IQR 3.73–5.18)] compared to PLWH without ART [3.40 mmol/L (IQR 2.90 – 4.10)] at adjusted *p* < 0.001 (**Table 1**). Total cholesterol levels were also significantly lower in PLWH without ART compared to PNLWH [4.15 mmol/L (IQR 3.60 – 4.88)] at adjusted *p* = 0.006.

The CRP levels were similar among the three study groups, whereas fibrinogen [*H* (2) = 8.11, *p* = 0.017] and D-dimer levels [*H* (2) = 9.88, *p* = 0.007] were significantly different (**Table 1**). Further analysis revealed that fibrinogen levels of PLWH without ART [0.41 ng/mL (IQR 0.17 – 1.00)] were significantly higher compared to both PLWH on ART [0.17 ng/mL (0.07 – 0.40)] at adjusted *p* = 0.025 and PNLWH [0.14 ng/mL (IQR 0.05 – 0.40)] at adjusted *p* = 0.031 (**Table 1**). D-dimer levels were significantly higher in PLWH on ART [41.54 ng/mL (IQR 17.10 – 176.92)] compared to PLWH without ART [17.50 ng/mL (IQR 2.19 – 57.80)] at adjusted *p* = 0.037 and PNLWH [15.18 ng/mL (IQR 3.69 – 96.32)] at adjusted *p* = 0.027.

### 3.2 Comparative analysis of the inflammatory and coagulation markers among distinct eGFR_cystC_ stages across the study groups

A comparative analysis of inflammatory markers across the different eGFR_cystC_ stages (CKD stages) were performed to evaluate the association between inflammation and the severity of renal dysfunction in the study population. The markers of inflammation and coagulation, including CRP, fibrinogen and D-dimer, were compared across the study groups according to the following eGFR_cystC_ stages, eGFR_cystC_ of ≥ 90 (normal renal function), eGFR_cystC_ of 60 – 89 (mild renal dysfunction), and eGFR_cystC_ of < 60 (CKD disease) (**Table 2**). There were no significant differences in median CRP levels among the eGFR_cystC_ stages in the overall population (*p* = 0.601), this was consistent with the overall PLWH cohort (*p* = 0.335), and PLWH on ART (*p* = 0.461) (**Table 2**). No significant differences were found in the median fibrinogen levels across the eGFR_cystC_ stages in the overall population (*p* = 0.641), overall PLWH (*p* = 0.850), and PLWH on ART (*p* = 0.969) (**Table 2**). In addition, no significant differences were observed between the two eGFR_cystC_ stages (eGFR_cystC_ of ≥ 90 and < 90) in the overall population and all study groups, for both CRP and fibrinogen (**Table 3**). D-dimer levels showed a significant difference among the three eGFR_cystC_ stages in the overall population [*H* (2) = 17.24, *p* < 0.001], overall PLWH [*H* (2) = 15.43, *p* < 0.001] and PLWH on ART [*H* (2) = 12.30, *p* = 0.002] (**Table 2**). Further analysis of the overall population revealed that median D-dimer levels were significantly higher in the eGFR_cystC_ of < 60 [159.56 ng/mL (IQR 21.42 – 270.24)] compared to eGFR_cystC_ of ≥ 90 [17.80 ng/mL (IQR 6.47 – 62.24)] at adjusted *p* = 0.027 and eGFR_cystC_ of 60–89 [91.56 ng/mL (IQR 42.46 – 236.55)] at adjusted *p* = 0.001 (**Table 2**). For the overall PLWH, median D-dimer levels were significantly higher in the eGFR_cystC_ of 60–89 [112.33 ng/mL (IQR 45.13 – 232.21)] compared to eGFR_cystC_ of ≥ 90 [25.44 ng/mL (IQR 7.27 – 55.43)] at adjusted *p* = 0.001 (**Table 2**). Further analysis of the PLWH on ART revealed that median D-dimer levels were significantly higher in the eGFR_cystC_ of < 60 [190.21 ng/mL (IQR 56.98 – 358.12)] compared to eGFR_cystC_ of ≥ 90 [29.49 ng/mL (IQR 13.45 – 87.42)] at adjusted *p* = 0.046 and eGFR_cystC_ of 60–89 [139.82 ng/mL (IQR 51.70 – 249.56)] at adjusted *p* = 0.009 (**Table 2**). Comparative analysis across the three eGFR stages for PLWH without ART was not feasible due to the limitation of the small sample size.

**Table 2:** Comparative analysis of markers of inflammation and coagulation among the three eGFR_cystC_ stages, including normal renal function, mild renal dysfunction and chronic kidney disease across the study groups.

	Overall population			
	eGFR_cystC_ ≥ 90	eGFR_cystC_ 60–89	eGFR_cystC_< 60	*p*-value
CRP (ng/mL)	0.78 (0.25–2.17)	0.71 (0.25–2.04)	0.50 (0.15–1.59)	0.601
Fibrinogen (ng/mL)	0.20 (0.06–0.50)	0.17 (0.09–0.34)	0.24 (0.08–6.76)	0.641
D-dimer (ng/mL)	17.80 (6.47–62.24)	91.56 (42.46–236.55)	159.56 (21.42–270.24)	<0 001
Overall PLWH				
CRP (ng/mL)	1.03 (0.27–3.93)	0.61 (0.21–2.74)	0.69 (0.14–1.68)	0.335
Fibrinogen (ng/mL)	0.30 (0.07–0.56)	0.17 (0.09–0.31)	0.22 (0.08–1.04)	0.850
D-dimer	25.44 (7.27–55.43)	112.33 (45.13–232.21)	172.13 (15.49–314.18)	< 0.001
PLWH on ART				
CRP (ng/mL)	1.09 (0.27–4.89)	0.61 (0.21–3.25)	0.59 (0.12–1.76)	0.461
Fibrinogen (ng/mL)	0.18 (0.04–0.41)	0.17 (0.08–0.31)	0.19 (0.08–0.35)	0.969
D-dimer	29.49 (13.45–87.42)	139.82 (51.70–249.56)	190.21 (56.98–358.12)	0.002

For the overall population, D-dimer is significant for eGFR_cystC_ of ≥ 90 versus eGFR_cystC_ of 60–89 (adjusted *p* = 0.001) and eGFR_cystC_ of < 60 (adjusted *p* = 0.027). For overall PLWH, D-dimer is significant for eGFR_cystC_ of ≥ 90 versus eGFR_cystC_ of 60–89 (adjusted *p* = 0.001). For PLWH on ART, D-dimer is significant for eGFR_cystC_ of ≥ 90 versus eGFR_cystC_ of 60–89 (adjusted *p* = 0.009) and eGFR_cystC_ of < 60 (adjusted *p* = 0.046). Results are expressed as median (interquartile range). ART, antiretroviral therapy; CRP, C-reactive protein; eGFR_cystC_, cystatin C-based estimated glomerular filtration rate; PLWH, people living with HIV. *p*-values in bold indicate significance.

**Table 3:** Markers of inflammation and coagulation between eGFR_cystC_ of ≥ 90 (normal renal function) and eGFR_cystC_ of < 90 (reduced renal function) across the study groups.

	Overall population		
	eGFR_cystC_ ≥ 90	eGFR_cystC_ < 90	*p*-value
CRP (ng/mL)	0.78 (0.25–2.17)	0.70 (0.20–1.73)	0.436
Fibrinogen (ng/mL)	0.20 (0.06–0.50)	0.18 (0.08–0.41)	0.819
D-dimer	17.80 (6.47–62.24)	142.83 (38.29–230.73)	< 0.001
Overall PLWH			
CRP (ng/mL)	1.03 (0.27–3.93)	0.65 (0.20–1.84)	0.150
Fibrinogen (ng/mL)	0.30 (0.07–0.56)	0.18 (0.08–0.36)	0.695
D-dimer	25.44 (7.27–55.43)	142.83 (44.69–230.73)	< 0.001
PLWH on ART			
CRP (ng/mL)	1.09 (0.27–4.89)	0.61 (0.19–1.85)	0.258
Fibrinogen (ng/mL)	0.18 (0.04–0.41)	0.17 (0.08–0.31)	0.920
D-dimer	29.49 (13.45–87.42)	152.02 (51.70–249.56)	< 0.001
PLWH without ART			
CRP (ng/mL)	1.01 (0.38–2.91)	0.69 (0.14–1.82)	0.272
Fibrinogen (ng/mL)	0.46 (0.17–0.88)	0.18 (0.17–12.81)	0.747
D-dimer	15.68 (1.95–47.05)	36.20 (10.56–172.27)	0.148

Results are expressed as median (interquartile range). ART, antiretroviral therapy; CRP, C-reactive protein; eGFR_cystC_, cystatin C-based estimated glomerular filtration rate; HIV, human immunodeficiency virus; PLWH, people living with HIV. *p*-values in bold indicate significance.

The D-dimer levels also displayed significant differences between eGFR_cystC_ of ≥ 90 and eGFR_cystC_ of < 90 for the overall population (U = 2404.50, *p* < 0.001), overall PLWH (U = 1338.00, *p* < 0.001) and PLWH on ART (U = 755, *p* < 0.001) (**Table 3**). Further analysis revealed that median D-dimer levels were significantly higher in reduced renal function [142.83 ng/mL (IQR 38.29 – 230.73)] compared to normal renal function [17.80 ng/mL (IQR 6.47 – 62.24)] for the overall population (**Table 3**). Moreover, median D-dimer levels were significantly higher in reduced renal function [142.83 ng/mL (IQR 44.69 – 230.73)] compared to normal renal function [25.44 ng/mL (IQR 7.27 – 55.43)] (**Table 3**). For PLWH on ART, median D-dimer levels were also significantly higher in reduced renal function [152.02 ng/mL (IQR 51.70 – 249.56) compared to normal renal function [29.49 ng/mL (IQR 13.45 – 87.42)] (**Table 3**). No difference in D-dimer levels was observed between the two eGFR_cystC_ stages for PLWH without ART (*p* = 0.148) (**Table 3**).

### 3.3 Association between inflammation, coagulation and renal function

#### 3.3.1 Spearman associations between inflammatory and renal function markers across the study groups

A Spearman rank correlation was performed to investigate the association between inflammatory and coagulation markers, and eGFR_cystC_ to assess the impact of chronic inflammation and coagulation on driving renal function decline in PLWH (**Supplementary Table 1**). Spearman correlation analysis revealed no significant associations between CRP and eGFRcystC across any of the study groups, including the overall PLWH cohort, those on ART, those not receiving ART, and PNLWH. Similarly, fibrinogen levels were not significantly correlated with renal function in any group. In contrast, D-dimer demonstrated a significant negative correlation with eGFR_cystC_ in the overall PLWH cohort (r = -0.26, *p* = 0.002) and more strongly among PLWH on ART (r = -0.36, *p* = 0.003), indicating that higher D-dimer levels were associated with reduced renal function. No such association was observed in PLWH not receiving ART or in PNLWH.

#### 3.3.2 Linear regression analysis for associations of inflammatory and coagulation with eGFR_cystC_ across the study groups

**Figure 2:**
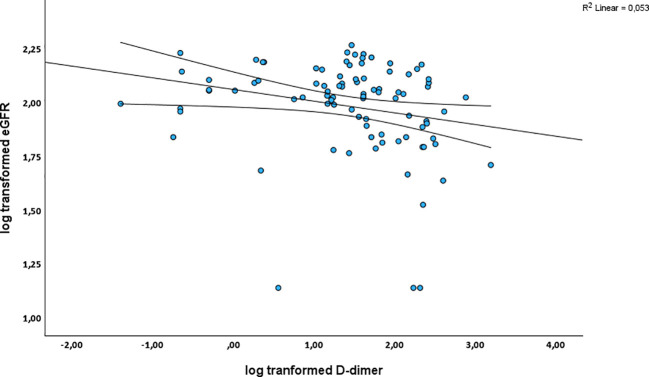
Scatter plot shows the inverse relationship between D-dimer levels and eGFR_cystC_ in the overall PLWH cohort, indicating that higher D-dimer levels are associated with reduced renal function. Straight line represents best fit/regression line while curved lines indicate mean confidence intervals.

A multiple linear regression was performed to evaluate the independent contribution of chronic inflammation and coagulation in driving renal function decline in PLWH. **Tables 4**, **5** show the multiple linear regression analysis between the inflammatory and coagulation markers and eGFR_cystC_ for the overall PLWH and PLWH on ART. Linear regression analysis was not carried out for PLWH without ART due to the limitation of the small sample size. In the overall PLWH, CRP was not associated with eGFR_cystC_ decline in both crude (*p* = 0.606) and adjusted analyses (*p* = 0.723), where age, tobacco use, BMI, glucose, blood pressure, and total cholesterol were included as covariates (**Table 4**). Fibrinogen was significantly associated with eGFR_cystC_ decline in the overall PLWH after adjusting for confounders [β = -1.34 (95% CI 2.68; - 0.01), *p* = 0.048)] (**Table 4**). D-dimer was significantly associated with eGFR_cystC_ decline in both crude [β= -0.06, (95% CI -0.09; -0.02), *p* = 0.004] and adjusted analysis [β = - 0.05 (95%CI - 0.08; - 0.01), *p* = 0.014] in the overall PLWH (**Table 4**). This relationship is further illustrated in **Figure 2**.

**Table 4:** Multiple linear regression analysis of inflammation and coagulation with eGFR_cystC_ in the overall cohort of people living with HIV.

Crude (R^2^ = 0.10, *p* = 0.008)			
	β	*P*-value	95% CI (Lower bound; upper bound)
CRP	-0.08	0.606	-0.38; 0.22
Fibrinogen	-1.34	0.064	-2.76; 0.08
D-dimer	-0.06	0.004	-0.09; -0.02

Adjusted (R^2^ = 0.25, *p* < 0.001)			
	β	*p*-value	95% CI (Lower bound; upper bound)
CRP	0.06	0.723	-0.25; 0.36
Fibrinogen	-1.34	0.048	-2.68; -0.01
D-dimer	-0.05	0.014	-0.08; -0.01

Factors adjusted include age, tobacco use, BMI, glucose, blood pressure, and total cholesterol. β, beta weight/correlation coefficient; CI, confidence interval; CRP, C-reactive protein; eGFR_cystC_, cystatin C-based estimated glomerular filtration rate. *p-*values in bold indicate significance.

**Table 5:** Multiple linear regression analysis of the independent association between inflammation and renal function in people living with HIV on antiretroviral therapy.

Crude (R^2^ = 0.10, *p* = 0.023)			
	β	*p-*value	95% CI (Lower bound; upper bound)
CRP	-0.09	0.566	-0.38; 0.21
Fibrinogen	-1.26	0.107	-2.81; 0.28
D-dimer	-0.05	0.009	-0.09; -0.01

Adjusted (R^2^ = 0.30, *p* < 0.001)			
	β	*p*-value	95% CI (Lower bound; upper bound)
CRP	0.07	0.647	-0.23; 0.37
Fibrinogen	-1.54	0.031	-2.92; -0.15
D-dimer	-0.06	0.004	-0.09; -0.02

Factors adjusted include age, tobacco use, BMI, glucose, blood pressure, and total cholesterol. β, beta weight/correlation coefficient; CRP, C-reactive protein; eGFR_cystC_, cystatin C-based estimated glomerular filtration rate. *p*-values in bold indicate significance.

**Figure 3:**
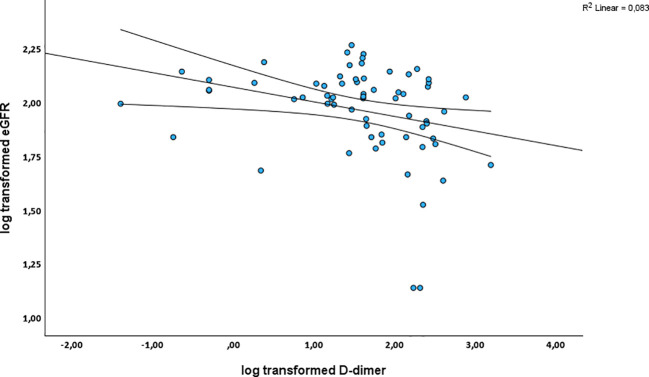
Scatter plot shows the inverse relationship between D-dimer levels and eGFR_cystC_ in PLWH on ART, indicating that higher D-dimer levels are associated with reduced renal function. Note: Straight line represents best fit/regression line while curved lines indicate confidence intervals.

When the data was analyzed for PLWH on ART, CRP was found not to be associated with eGFR_cystC_ before (*p* = 0.566) and after adjustment (*p* = 0.647) (**Table 5**). Fibrinogen was significantly associated with eGFR_cystC_ decline after adjusting for confounders [β = -1.54, (95% CI - 2.92; - 0.15), *p* = 0.031] (**Table 5**). D-dimer was significantly associated with eGFR_cystC_ decline in both the crude [β = -0.05, (95%CI - 0.09; -0.01), *p* = 0.009] and adjusted analysis [β = -0.06, (95% CI - 0.09; - 0.02), *p* = 0.004] (**Table 5**). This relationship is further illustrated in **Figure 3**.

### 3.4 Binary logistic regression analysis predicting the odds of developing chronic kidney disease across the study groups

Further analysis using binary logistic regression was performed to evaluate if higher levels of inflammatory (CRP) and coagulation markers (fibrinogen or D-dimer) were associated with the increased odds of developing CKD (**Table 6**). After adjustment, fibrinogen was significantly associated with eGFR_cystC_ of < 60, with an odds ratio of 1.12 (95% CI 1.00 – 1.23, *p* = 0.041) in the overall PLWH cohort (**Table 6**). In PLWH on ART, D-dimer was also significantly associated with eGFR_cystC_ of < 60 after adjusting for confounders, with an odds ratio of 1.01 (95% CI 1.00 – 1.03, *p* = 0.045) (**Table 6**).

**Table 6:** Binary logistic regression analysis predicts the risk of developing chronic kidney disease in the overall cohort of people living with HIV and PLWH on antiretroviral therapy.

	Overall PLWH			
	eGFR_cystC_ < 60		eGFR_cystC_ < 60	
	OR (95% CI)	*p*-value	AOR (95% CI)	*p*-value
CRP	0.80 (0.49; 1.31)	0.379	0.87 (0.59;1.29)	0.480
Fibrinogen	1.09 (0.99; 1.20)	0.058	1.12 (1.00; 1.23);	0.041
D-dimer	1.00 (1.00; 1.01)	0.066	1.01 (0.00; 1.01)	0.177

	PLWH on ART			
	eGFR_cystC_ < 60		eGFR_cystC_ < 60	
	OR (95% CI)	*p*-value	AOR (95% CI)	*p*-value
CRP	0.67 (0.29; 1.53)	0.341	0.30 (0.02; 3.68)	0.345
Fibrinogen	1.16 (0.97; 1.38)	0.112	1.40 (0.95; 2.07)	0.094
D-dimer	1.00 (1.00; 1.01)	0.087	1.01 (1.00; 1.03)	0.045

Factors adjusted include age, tobacco use, BMI, blood pressure, glucose and total cholesterol. Abbreviations: AOR: adjusted odds ratio; CI: confidence interval; CRP: C-reactive protein; eGFR_cystC_: cystatin C-based estimated glomerular filtration rate; OR: odds ratio. *p*-values in bold indicate significance.

## 4 Discussion

This study investigated the association between systemic inflammation, coagulation activation, and renal function among people living with HIV (PLWH), particularly those receiving antiretroviral therapy (ART). The findings demonstrate that coagulation markers, D-dimer and fibrinogen, were independently associated with renal function decline and increased risk of chronic kidney disease (CKD). In contrast, the inflammatory marker CRP showed no significant association with renal function across the study groups. Notably, D-dimer showed consistent and stronger associations with declining eGFR_cystC_, especially among PLWH on ART, suggesting that coagulation activation may play a more prominent role than systemic inflammation in renal dysfunction in this population.

Our initial results revealed that while ART did not affect CRP levels in PLWH, its impact on fibrinogen and D-dimer was distinct. Fibrinogen levels were elevated in PLWH not on ART, whereas D-dimer levels remained persistently high even in those receiving treatment. Our findings indicate that ART did not demonstrate a significant suppressive effect on markers of inflammation in treated PLWH compared to PNLWH. Concurrently, coagulation markers remained elevated in the ART-treated cohort. This persistent elevation in coagulation could potentially be attributed to ongoing immune activation or specific effects associated with certain ART regimens (48–51). Human immunodeficiency virus infection is associated with chronic inflammation through activation of innate immunity by causing depletion of the mucosal CD4+ T-cells, which alters the integrity of the mucosal barrier (52, 53). This does not exclude the fact that PLWH on ART may continue to experience a chronic state of low-grade inflammation, as has been well documented (33). Interestingly, the results also indicate that the state of coagulation activation in treated PLWH may persist, amplified by some ART regimens, which supports the current literature that coagulation decreases significantly in early ART (first months) but persists with long-term treatment (11, 15, 54, 55). The majority of PLWH in this study were on long-term ART, with a median duration being 53 months, which may explain this finding. Some studies have linked protease inhibitor) use such as lopinavir/ritonavir, and abacavir use to increased coagulation activation through metabolic or endothelial side effects (17, 49), which may partially explain our finding as a considerable portion of PLWH were on abacavir and PI-based therapy as showed by **Figure 1**. This state of chronic inflammation and increased coagulation activation may predispose individuals to increased risk of adverse events such deteriorated renal function.

When CRP was compared between the various stages of eGFR_cystC_, the findings revealed no significant differences, suggesting no clear association between systemic inflammation and the severity of renal dysfunction in PLWH. These results are partially consistent with a previous study that also failed to establish a strong correlation between CRP and early-stages of CKD in PLWH (56), however, they contrast with reports indicating elevated inflammatory markers as predictors of renal impairment, particularly in more advanced diseases or in cohorts with additional co-morbidities (13, 15, 57, 58). Notably, other research groups like Gupta et al. (14) have seen no association between CRP and eGFR in PLWH when the chronic kidney disease epidemiology (CKD-EPI) collaboration equation was used. One possible explanation for the lack of differences within our cohort could be the relatively young population, early CKD stages, or effective ART-mediated viral suppression, reducing systemic inflammatory burden (33, 59, 60). This study revealed no linear association between inflammation and renal function decline among PLWH, even after adjusting for confounders such as age, tobacco use, BMI, glucose, blood pressure, and total cholesterol. These results suggest that chronic inflammation may not be linked to gradual renal function decline among PLWH. The results further showed that elevated CRP was not a predictor of CKD, indicating no direct involvement of chronic inflammation in CKD risk and progression in this cohort of PLWH. These findings contradict previous work implicating a chronic state of inflammation in deteriorated renal function and CKD progression among the general population and PLWH (13, 61, 62). The mechanisms of renal function decline may be more complex in PLWH. Other factors such as the direct effects of specific cytotoxic ARV drugs, co-morbidities and more specific inflammatory markers may play a more significant role.

Notably, D-dimer levels were significantly elevated in mild renal dysfunction for the overall PLWH and remained persistently elevated throughout declining eGFR_cystC_ stages for those on treatment, suggesting an increase in coagulation activation with the severity of renal dysfunction among PLWH, particularly on ART. The results showed a consistent pattern of independent association between the elevated coagulation marker levels (fibrinogen and D-dimer) and progressive renal function decline from the overall PLWH cohort to those exclusively on ART treatment. These results indicate that an increase in coagulation activation may be linked to gradual loss of renal function in this vulnerable population of PLWH on ART. Further analysis with logistic regression has revealed elevated coagulation markers (fibrinogen and D-dimer) as independent predictors of CKD, suggesting the role of enhanced coagulation activation as a major driver of CKD advancement among PLWH. This also affirms the initial results highlighting the contribution of this pathological process to progressive renal function decline, which is possibly mediated by immune activation/chronic inflammation or ART-related toxic effects. These findings corroborate previous work linking persistent coagulation activation to renal marker elevation or renal dysfunction in PLWH (15, 62, 63). Moreover, the stronger association observed between coagulation markers (particularly D-dimer) and renal dysfunction suggests a mechanism linked to endothelial injury and microvascular compromise in PLWH (49). HIV infection, even in virally suppressed individuals, is known to amplify tissue factor expression and thrombin generation, leading to subclinical hypercoagulability (21). This pathway may be more directly linked to glomerular dysfunction than systemic inflammation markers such as high-sensitivity C-reactive protein or interleukin 6, which may be more reflective of acute or generalized immune activation rather than renal injury. In contrast to prior studies that reported parallel elevations in inflammatory markers, our findings indicate a more specific coagulation-dominant phenotype, suggesting that inflammation and coagulation may be partially uncoupled in ART-treated PLWH.

The stronger association observed for D-dimer and fibrinogen compared to CRP may be explained by their closer involvement in pathways directly linked to renal injury. D-dimer reflects ongoing fibrin formation and degradation, indicating heightened thrombin generation and fibrinolytic activity, while fibrinogen contributes to increased blood viscosity and clot formation (64, 65). In PLWH, persistent immune activation and ART-related endothelial dysfunction may promote tissue factor expression, leading to a hypercoagulable state (66, 67). This can result in microvascular thrombosis and impaired renal perfusion, ultimately contributing to glomerular injury and progressive decline in kidney function (68). In contrast, CRP is a general marker of systemic inflammation and may not adequately capture localized vascular or renal-specific pathological processes, particularly in treated PLWH. Long-term ART exposure, particularly with certain regimens, may further contribute to endothelial dysfunction and metabolic disturbances, thereby sustaining coagulation activation despite viral suppression (66).

The current findings highlight the potential of coagulation markers, particularly D-dimer, as indicators for early and progressive renal impairment in PLWH on ART. This study has several limitations. First, its cross-sectional design precludes causal inference, and therefore the observed associations cannot establish temporal relationships between coagulation activation and renal dysfunction. Second, the relatively small sample size, particularly among PLWH without ART, may limit statistical power and generalizability. Third, we were unable to adjust for important clinical variables such as ART regimen type and CD4+ count, which may influence both coagulation and renal outcomes. Additionally, the use of a limited panel of inflammatory markers, particularly reliance on CRP alone, may not fully capture the complexity of immune activation in PLWH. Future longitudinal studies incorporating broader inflammatory and endothelial biomarkers are needed to better elucidate these relationships. Despite these limitations, the study provides vital preliminary information on the implications of coagulation activation in contributing to CKD among PLWH receiving ART, which may serve as a guide for future work. Future studies should investigate longitudinal changes in coagulation and renal biomarkers to determine whether these markers can predict early renal dysfunction in PLWH.

## 5 Conclusion

The present study has revealed that PLWH on ART may have increased levels of coagulation markers, particularly D-dimer, which is independently associated with renal function decline and risk of CKD. These results underscore the potential value of D-dimer and fibrinogen as early indicators of renal function decline and CKD in this population. The markers of coagulation, particularly D-dimer could be valuable in risk stratification and monitoring strategies for kidney health in this vulnerable population. Future longitudinal research should clarify the causal pathways to confirm the importance of monitoring coagulation markers despite long-term viral suppression as a tool to prevent CKD progression and improve outcomes among PLWH receiving ART.

## Data Availability

The original contributions presented in the study are included in the article/**Supplementary Material**. Further inquiries can be directed to the corresponding authors.
