# Supplementary material for: Coagulation is associated with renal function decline and chronic kidney disease in people living with HIV in South Africa
Source: Front Nephrol. 2026 May 22;6:1800778. doi: 10.3389/fneph.2026.1800778 (PMC13236642; doi:10.3389/fneph.2026.1800778)
Supplement: Supplementary file 1 [file DataSheet1.pdf]

Quality assurance and control procedures for biochemical tests:

All biochemical analyses were performed in accordance with the manufacturers' protocols and standard laboratory operating procedures. The Luminex® xMAP® system and Cobas Integra® 400 Plus autoanalyzer were calibrated prior to sample analysis using manufacturer-provided calibration standards. Internal quality control samples at low, medium, and high concentrations were included in each analytical run to ensure assay precision and accuracy. All samples were analysed in duplicate, and variability between replicates was monitored to ensure consistency.

Standard curves were generated for each analyte and validated across kit lots prior to analysis. Assay performance, including sensitivity, specificity, and intra-assay variability, was verified according to manufacturer specifications. In addition, sample preparation, handling, and storage were conducted under controlled conditions to minimise pre-analytical variability.

External quality assurance was maintained through adherence to institutional laboratory standards and participation in routine laboratory quality monitoring procedures. All analyses were conducted under expert supervision to ensure compliance with established protocols and reliability of results.
