# Supplementary material for: Coagulation is associated with renal function decline and chronic kidney disease in people living with HIV in South Africa
Source: Front Nephrol. 2026 May 22;6:1800778. doi: 10.3389/fneph.2026.1800778 (PMC13236642; doi:10.3389/fneph.2026.1800778)
Supplement: Supplementary file 2 [file Table1.docx]

**Supplementary Table 1**. Spearman rank associations of markers of inflammation, and coagulation with eGFR_cystC_ across the study groups.

| Overall PLWH | | | |
| --- | --- | --- | --- |
|  | **Correlation coefficient (r)** | ***p*-value** | **95% CI (lower bound; upper bound)** |
| CRP-eGFR_cystC_ | 0.18 | 0.094 | -0.04; 0.38 |
| Fibrinogen- eGFR_cystC_ | 0.12 | 0.256 | -0.09; 0.32 |
| D-dimer- eGFR_cystC_ | -0.26 | **0.002** | -0.42; -0.09 |
| PLWH on ART | | | |
| CRP-eGFR_cystC_ | 0.16 | 0.191 | -0.09; 0.40 |
| Fibrinogen- eGFR_cystC_ | 0.07 | 0.561 | -0.18; 0.32 |
| D-dimer- eGFR_cystC_ | -0.36 | **0.003** | -0.56; -0.12 |
| PLWH without ART | | | |
| CRP-eGFR_cystC_ | 0.26 | 0.210 | -0.163; 0.602 |
| Fibrinogen- eGFR_cystC_ | 0.05 | 0.826 | -0.366; 0.443 |
| D-dimer- eGFR_cystC_ | -0.078 | 0.712 | -0.47; 0.34 |
| PNLWH | | | |
| CRP-eGFR_cystC_ | 0.16 | 0.315 | -0.16; 0.45 |
| Fibrinogen- eGFR_cystC_ | -0.16 | 0.323 | -0.45; 0.16 |
| D-dimer- eGFR_cystC_ | -0.13 | 0.403 | -0.43; 0.19 |

Abbreviations: ART: antiretroviral therapy; CI: confidence interval; CRP: C-reactive protein; eGFR_cystC_: cystatin C-based estimated glomerular filtration rate; HIV: human immunodeficiency virus; PLWH: people living with HIV. PNLWH: people not living with HIV. *p*-values in bold indicate significance.
